# Changes in sleep architecture during recurrent cycles of sleep restriction: a comparison between stable and variable short sleep schedules

**DOI:** 10.1093/sleepadvances/zpaf016

**Published:** 2025-03-15

**Authors:** Tiffany B Koa, Ju Lynn Ong, June C Lo

**Affiliations:** Centre for Sleep and Cognition and Human Potential Translational Research Programme, Yong Loo Lin School of Medicine, National University of Singapore, Singapore; Centre for Sleep and Cognition and Human Potential Translational Research Programme, Yong Loo Lin School of Medicine, National University of Singapore, Singapore; Centre for Sleep and Cognition and Human Potential Translational Research Programme, Yong Loo Lin School of Medicine, National University of Singapore, Singapore

**Keywords:** sleep architecture, intraindividual sleep variability, partial sleep deprivation, recovery sleep, slow wave activity

## Abstract

**Study Objectives:**

To examine how sleep architecture changes over successive cycles of restricted and recovery sleep in young adults, and to determine whether sleep-restricted schedules with differing night-to-night variability in sleep durations lead to different sleep physiological responses.

**Methods:**

In this 15-night laboratory-based study, 52 healthy young adults (25 males, age: 21–28) were randomly assigned to one of three sleep schedules: stable short, variable short, or control. They underwent two baseline nights of 8-h time-in-bed (TIB), followed by two cycles of “weekday” sleep opportunity manipulation and “weekend” recovery (8-h TIB). During each manipulation period, the stable short sleep and the control groups received 6-h and 8-h TIBs each night, respectively, while the variable short sleep group received 8-h, 4-h, 8-h, 4-h, and 6-h TIBs from the first to the fifth night. Polysomnography was conducted every night.

**Results:**

Sleep architecture changes induced by both short sleep schedules returned to baseline levels following the first or second recovery night and were largely similar between the first and second periods of sleep restriction. Sleep parameters averaged across each sleep restriction or recovery period showed no significant differences between the two short sleep groups.

**Conclusions:**

The similar sleep physiological responses in the two sleep restriction periods suggest that in young adults, sleep architecture does not adapt to recurrent weeks of moderate partial sleep loss, and such sleep patterns did not have compounding effects on sleep architecture. Furthermore, overall, increasing night-to-night variability in sleep duration did not have much additional impact on sleep physiological responses relative to a stable short sleep schedule.

**Clinical Trial:**

Performance, Mood, and Brain and Metabolic Functions During Different Sleep Schedules (STAVAR), https://www.clinicaltrials.gov/study/NCT04731662, NCT04731662

**Statement of Significance**

During recurrent cycles of restricted and recovery sleep, young adults following schedules with different sleep duration variability on average exhibit similar sleep physiological responses across the sleep restriction periods. This suggests that adopting such short sleep schedules repeatedly may not aggravate the effects on sleep architecture in this age group, and that sleep duration variability may be a negligible factor as compared to overall sleep duration. Sleep loss-induced changes to sleep architecture are restored quickly with 1–2 nights of recovery sleep, starkly contrasting with neurobehavioral responses that not only show limited recuperation but may also compound with re-exposure to sleep restriction.

## Introduction

Insufficient sleep is a pervasive issue in many industrialized societies, with up to 74% of adults getting less than 7 h of sleep [1–3]—the minimum duration recommended by the National Sleep Foundation for this age group [4]. Numerous studies have shown that multiple nights of sleep restriction can lead to cumulative impairments in a wide range of neurobehavioral functions [5], such as sustained attention, processing speed, alertness, and affect [6–10], and that ensuing nights of recovery sleep may only offer limited, if any, alleviation of these deficits [6, 11, 12].

Several studies have examined how sleep curtailment impacts sleep architecture and the subsequent recovery dynamics. Existing research has shown that multiple nights of partial sleep loss result in reduced durations of N1, N2, and rapid eye movement (REM) sleep [6, 10, 11, 13–17], while slow wave sleep (SWS) duration is generally preserved [6, 10, 14, 15]. Some studies have also reported reduced sleep onset latency and wake after sleep onset (WASO) [16, 17], while sleep efficiency increased [11, 17]. Additionally, slow wave activity (SWA) increases upon exposure to sleep restriction but quickly stabilizes thereafter [15, 18]. During subsequent recovery sleep periods, most studies in adults show that sleep architecture returns to baseline levels within 1–3 recovery nights [6, 11, 13, 15], with the recovery time varying across studies in part due to the differing severity and number of nights of sleep restriction.

A limitation of most of these previous studies is that they only focused on a single cycle of sleep restriction and recovery, and only a few have addressed sleep physiological responses when individuals are re-exposed to another period of sleep restriction. We previously investigated how sleep architecture changed in repeated cycles of sleep restriction and extension in adolescents [19]. We found that following five nights of 5-h time-in-bed (TIB), one night of 9-h recovery sleep was unable to restore durations of non-rapid eye movement (NREM) sleep, REM, WASO, N2 latency, and sleep efficiency to baseline levels, and that even with two nights of recovery sleep, some of the changes in sleep architecture, or more specifically, increased sleep efficiency and shorter sleep onset latency, still became more pronounced when participants were re-exposed to another round of sleep restriction. On the other hand, one recent study seems to suggest that adults’ sleep physiological responses to recurrent cycles of sleep restriction and recovery are different. Banks et al. [20] had adult participants undergo two periods of five-night sleep restriction (4-h TIB nightly), separated by a single intervention night that had TIBs ranging from 0 h to 12 h. With the polysomnographic data collected on selected nights, e.g. the beginning and the end of each sleep restriction period, they found that total sleep time (TST), N2 and REM sleep durations, as well as slow wave energy, remained stable across both sleep restriction periods and showed a dose–response effect on the intervention night such that longer durations of TST, N2, and REM sleep were observed with longer TIB, while SWS was preserved throughout the protocol except for when TIB was less than 4 h. Thus, unlike with adolescents, repeated cycles of sleep restriction may have little to no additional effect on sleep architecture changes in adults. The first aim of the current study is therefore to examine the changes in sleep architecture over successive cycles of sleep restriction on simulated weekdays and recovery sleep on simulated weekends in young adults. The present study would also seek to expand on Banks et al.’s findings by more closely tracking sleep architecture changes on a nightly basis (instead of just at the start and end of each sleep restriction period).

Additionally, whether the limited differences in sleep physiological responses during recurrent sleep loss can be generalized to a more variable short sleep schedule is unknown because sleep restriction in existing experimental studies is usually distributed evenly across nights, e.g. 4-h TIB each night. In real life, while many individuals extend their sleep on weekends, it should be noted that some people also catch up on sleep on weekdays, and such mid-week sleep extensions, which are seemingly more common among people in Asian countries [21], increase the night-to-night variability in sleep duration.

The effects of night-to-night sleep duration variability on sleep architecture have not been well studied, and the few studies conducted thus far have yielded inconsistent findings. One study by Taub [22] found that during two nights of 8-h TIB, participants with greater sleep duration variability had, on average, significantly less stage 4 and REM sleep, but more stage 2 sleep than regular sleepers. This is in contrast with a more recent study by Sun et al. [23] comparing between a stable sleep group that received 7.5 h TIB nightly and a variable sleep group that received alternating TIBs of 6 h and 9 h over 6 manipulation nights. No significant differences were found in most of the sleep parameters (average N1, SWS, and REM sleep durations, sleep onset latency, and WASO) between the variable and stable sleep groups, except for average N2 duration, which was about 22 min longer in the variable than the stable sleep group. The largely similar sleep macrostructure between variable and stable sleep schedules was also found in a study in which participants switched between stable and variable sleep schedules with comparable average sleep lengths of 7.4–7.5 h [24]. It should also be noted that these studies investigated sleep schedules with average nightly TIBs within the recommended range of 7–9 h. It is unclear if findings will be similar when average nightly TIBs are curtailed. The second aim of the study is therefore to determine whether two sleep-restricted schedules with differing amounts of sleep duration variability (some vs. none) across the simulated weekdays would lead to different changes in sleep architecture as compared to a well-rested sleep schedule. Such a direct comparison would enable us to establish which, if any, would be a better schedule for individuals who regularly get insufficient sleep on weekdays.

## Methods

### Participants

Fifty-nine young adults were recruited for this study. Participants met the following eligibility criteria: (1) were between 21 and 35 years of age, (2) had a body mass index (BMI) between 18.5 and 24.9 kg/m^2^, (3) did not suffer from sleep disorders, chronic physical illnesses, or mental disorders (based on self-report, not categorized as having a high risk of sleep apnea using the Berlin Questionnaire [25], fell within the normal range for all the subscales of the Depression, Anxiety and Stress Scale—21 items [26], Epworth Sleepiness Scale (ESS) [27] score <12, Insomnia Severity Index (ISI) [28] score <8, and Pittsburgh Sleep Quality Index (PSQI) [29] global score <6), (4) had a daily average TIB of at least 6 h according to their self-report and hence, were not habitual short sleepers, (5) did not have an extreme chronotype (Morningness–Eveningness Questionnaire [30] score: 31–69), (6) were not shift workers, (7) did not smoke, (8) did not typically consume more than 4 cups of caffeinated beverages per day, (9) did not typically consume more than 13 units of alcohol per week, and (10) did not travel across more than 2 time zones a month prior to the study. Full details about the recruitment process and additional inclusion criteria relevant to other parts of the study have previously been reported [8]. Informed written consent was obtained from all participants.

During the week preceding the experiment, participants had to follow an 8-h nocturnal sleep schedule to help entrain their circadian rhythms and to minimize any effects of prior sleep loss. They were prescribed a consistent wake time that matched their self-reported habitual wake time; this same wake time was then used during the experiment itself. Actigraphy and sleep diaries were used to monitor participants’ sleep and ensure compliance. During the 3 days prior to the study, participants were also restricted from napping or consuming any food and beverages that contained alcohol or caffeine.

Before the start of the experiment, four participants withdrew due to personal or medical reasons. Two participants who failed to adhere to their pre-experiment sleep schedules were dropped on day 1 of the study. One participant in the control group withdrew mid-study for personal reasons; the data were retained in the analyses as the participant had completed 13 out of the 15 nights of the protocol (86.67%). Data from another participant in the control group were excluded from analyses due to poor sleep throughout the protocol (sleep efficiency evaluated with polysomnography (PSG) was below 80% for 8 nights and reached a minimum of 67.6%). The final analyses thus included data from 52 participants.

All participants were financially reimbursed for their participation. This study was approved by the Institutional Review Board of the National University of Singapore.

### Protocol

**Figure 1.**
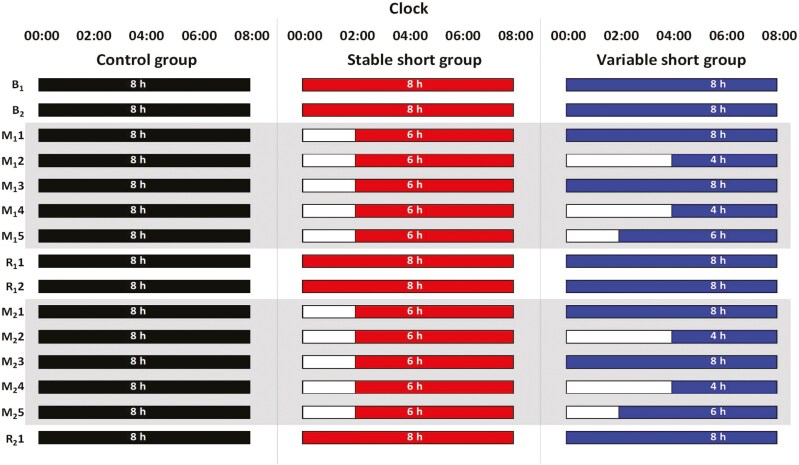
15-night experimental protocol. All groups started with an adaptation (B_1_) and a baseline night (B_2_) of 8-h TIB. These were followed by the first cycle of five nights of sleep opportunity manipulation (M_1_1–M_1_5) and two nights of recovery sleep (R_1_1 and R_1_2). The second cycle consisted of another five nights of sleep opportunity manipulation (M_2_1–M_2_5) and one night of recovery sleep (R_2_1). Wake times were aligned to each participant’s habitual wake times and were kept constant throughout the protocol, with shorter TIBs achieved by delaying bedtimes. Shaded gray areas indicate the sleep manipulation periods. Sleep timings illustrated here are based on a wake time of 08:00.

The study lasted 15 nights (Figure 1) and was conducted at the Centre for Sleep and Cognition, National University of Singapore. Each participant was allocated an individual, sound-attenuated, air-conditioned, windowless sleep room. Participants were not allowed to sleep outside of scheduled sleep periods and were continuously monitored by the research staff. Upon waking in the morning, participants were encouraged to spend their time in a common area which received natural and artificial light during the daytime (average: 287.50 lux). During the nighttime, participants were exposed to constant levels of artificial lighting until their bedtime (average: 183.42 lux). During their wake periods, participants were administered several cognitive tasks and questionnaires throughout the day, and results regarding their neurobehavioral functioning have been reported previously [8].

Participants were randomly assigned to one of three groups—the stable short sleep group (*n* = 19), the variable short sleep group (*n* = 18), and the control group (*n* = 15). All participants were provided with an 8-h sleep opportunity during the two baseline nights of the protocol (B_1_ and B_2_; Figure 1) for adaptation and baseline measurement purposes. Following this, participants underwent two cycles of sleep opportunity manipulation and recovery. Each cycle consisted of five nights of sleep opportunity manipulation and 1–2 subsequent nights of recovery sleep. During each manipulation period (M_1_1–M_1_5; M_2_1–M_2_5), the control group received an 8-h sleep opportunity for each of the five manipulation nights; the stable short sleep group received a 6-h TIB nightly, while the variable short sleep group had TIBs of 8 h, 4 h, 8 h, 4 h, and 6 h from the first to the fifth night. Both short sleep groups were therefore given the same total TIB duration (30 h) across the manipulation period of each cycle. During the recovery nights (R_1_1–R_1_2 and R_2_1), all participants received an 8-h TIB nightly. Throughout the entire protocol, participants were awoken at their habitual wake time; bedtimes were delayed accordingly for nights with shorter TIBs. Participants’ sleep was measured using PSG every night.

### PSG

Electroencephalography (EEG) was performed using a SOMNOtouch recorder (SOMNOmedics GmbH, Randersacker, Germany) on three channels—C3, F3, and O1 in the International 10–20 system, with the contralateral mastoid (A2) used as reference. On rare occasions, such as when a participant showed minor skin sensitivity at the electrode placement site following multiple days of PSG application, channels C4, F4, and O2 would be used instead, with A1 serving as the reference. Electrodes placed at Cz and Fpz were used as common reference and ground electrodes, respectively. Electrooculography (EOG) and submental electromyography (EMG) were also used. Impedance was kept below 5 kΩ for EEG and 10 kΩ for EOG and EMG electrodes. Signal was sampled at 256 Hz and filtered between 0.2 and 35 Hz for EEG and between 0.2 and 10 Hz for EOG. Pulse oximetry was used on the first night (B_1_) to screen for undiagnosed sleep apnea.

### Sleep staging and EEG spectral analysis

Sleep stages and artifactual epochs were automatically scored every 30 s using an automatic PSG scoring software [31] (Neurobit PSG, Neurobit Inc., NY, USA) in conjunction with the FASST toolbox [32] (http://www.montefiore.ulg.ac.be/~phillips/FASST.html), and visually checked by trained technicians, following criteria set by the American Academy of Sleep Medicine Manual for the Scoring of Sleep and Associated Events [33]. The Neurobit software utilized the C3/A2 (or C4/A1), EOG1/A2 and EOG2/A2 (or EOG1/A1 and EOG2/A1), and EMG input channels for sleep staging, whereas the trained technicians used all available channels (including O1/A2 and F3/A2) for scoring sleep. It should be noted that while the technicians were not informed of the group assignments while scoring, it is possible that they could infer this based on the duration of the PSG recordings. Since B_1_ night was intended for adaptation, polysomnographic recordings from that night were excluded from all the statistical analyses. Recordings with 10% or more epochs that could not be scored (13 out of 719 recordings) and recordings containing more than 10% artifacts from epochs scored as sleep (2 recordings) were also excluded from analyses. Thus, 704 recordings were included in the analyses.

The following parameters were derived for each recording: TST, N1 duration, N2 duration, SWS duration, REM duration, N2 latency, WASO, and sleep efficiency.

EEG spectral analysis was performed on non-overlapping 5-s epochs using custom routines written in Matlab R2012a (The MathWorks Inc., Natick, MA, USA). Analysis was conducted using C3/A2 (or C4/A1). For each epoch, power spectral density estimates were computed using Welch’s modified periodogram method [34] (Hamming window; 0.2-Hz bin resolution), and spectral power was computed from 0.6 to 4 Hz using the trapezoidal rule for integral approximation. Mean SWA (total SWA divided by duration spent in NREM sleep) was computed and expressed as a percentage relative to baseline values (B_2_).

### Statistical analysis

Statistical analyses were conducted using SAS software 9.4 (SAS Institute Inc., Cary, NC, USA). General linear mixed models with PROC MIXED were used to determine the effects of group, night, and group × night interaction on all the sleep parameters. The effect sizes, Cohen’s *ƒ*^2^, were calculated as *ƒ*^2^ = (*u*/*v*) × *F*, with *u* and *v*, respectively, being the numerator and denominator degrees of freedom of the *F* statistic [35, 36]. The cutoffs for small, medium, and large effect sizes were 0.02, 0.15, and 0.35, respectively [35].

Additionally, the average values across baseline (B: B_2_) and each period of sleep manipulation (M_1_: M_1_1–M_1_5 and M_2_: M_2_1–M_2_5) and recovery sleep (R_1_: R_1_1–R_1_2 and R_2_: R_2_1) were derived for all sleep parameters. To determine whether these average values showed any group differences over the five sleep periods (B, M_1_, R_1_, M_2_, and R_2_), general linear mixed models were again conducted.

## Results

Participants in the three groups did not show any significant difference in age, sex distribution, BMI, caffeinated drink and alcohol consumption, levels of depression, anxiety, or stress, levels of daytime sleepiness, symptoms of insomnia, morningness–eveningness preference, self-reported habitual sleep durations and timings, and PSQI global score (*p* > .11; Table 1).

**Table 1.** Demographics and habitual sleep characteristics for all groups

	Stable Short		Variable Short		Control			
	Mean	*SD*	Mean	*SD*	Mean	*SD*	*F*/χ^2^	*P*
*n*	19	–	18	–	15	–	–	–
Age (years)	22.68	1.77	22.72	1.67	22.87	1.73	0.05	.95
Sex (% males)	52.63	–	44.44	–	46.67	–	0.27	.88
BMI	20.85	1.61	20.43	2.08	21.76	2.19	1.96	.15
Caffeinated drinks per day (cups)	0.59	0.76	0.93	0.87	0.78	1.07	0.66	.52
Alcohol consumed per week (units)	0.81	1.21	0.59	1.67	0.83	1.82	0.12	.89
DASS-21								
Depression score	1.89	2.05	3.22	3.23	1.47	2.07	2.24	.12
Anxiety score	2.11	2.16	2.00	2.74	0.93	1.28	1.41	.25
Stress score	3.26	2.92	2.33	3.96	2.93	3.61	0.33	.72
ESS score	5.84	3.61	5.78	3.70	4.20	2.57	1.21	.31
ISI score	4.11	3.28	3.17	3.17	3.33	2.69	0.48	.62
MEQ score	47.21	5.66	45.11	6.43	48.53	8.28	1.09	.34
PSQI								
Average TIB (h)	7.88	0.63	8.03	0.65	8.11	0.99	0.41	.67
Average TST (h)	7.27	0.59	7.55	0.58	7.61	0.91	1.15	.32
Bedtime (clock time)	00:36	00:46	00:39	01:03	00:18	01:00	0.64	.53
Wake time (clock time)	08:29	00:56	08:41	01:02	08:25	01:09	0.31	.73
Global score	3.37	1.67	2.83	1.47	2.60	1.72	1.03	.36

BMI, body mass index; DASS-21, Depression, Anxiety and Stress Scale—21 items; ESS, Epworth Sleepiness Scale; ISI, Insomnia Severity Index; MEQ, Morningness–Eveningness Questionnaire; PSQI, Pittsburgh Sleep Quality Index; *SD*, standard deviation; TIB, time-in-bed; TST, total sleep time.

**Figure 2.**
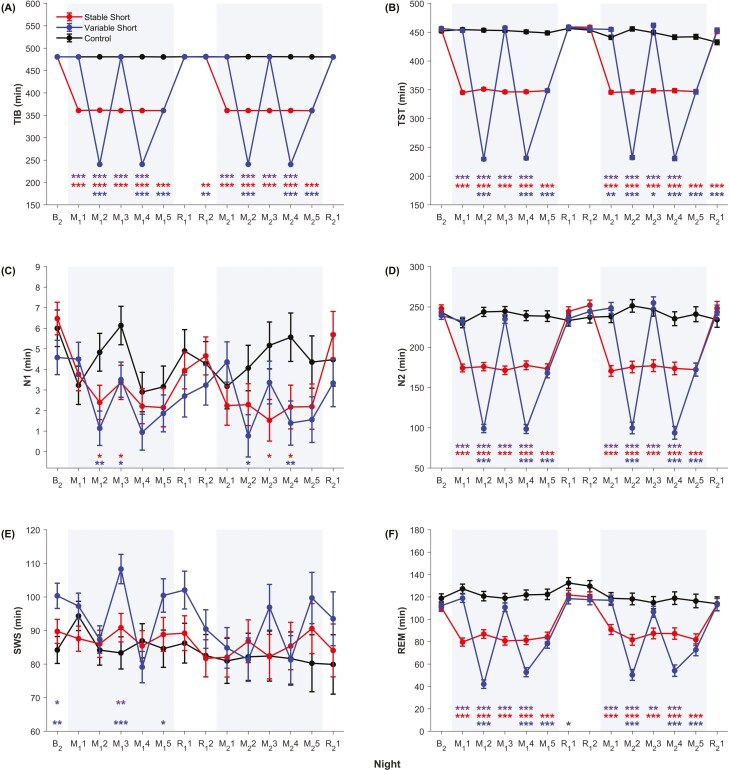

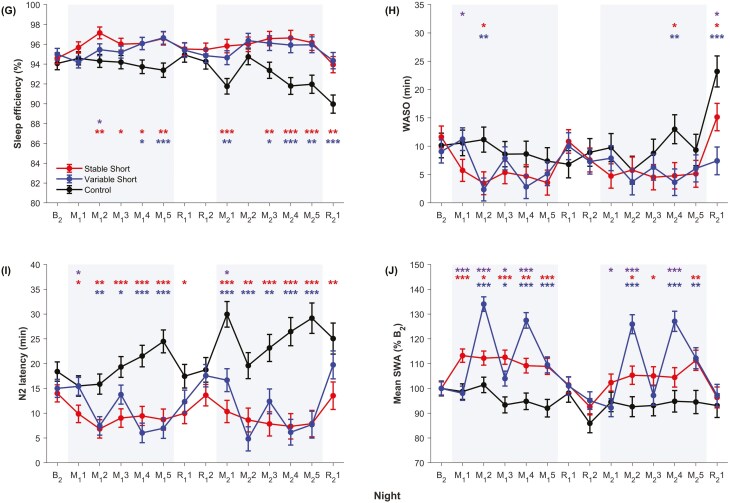
Effects of stable and variable short sleep on sleep across study nights. Means and standard errors derived from general linear mixed models are plotted for (A) time-in-bed (TIB), (B) total sleep time (TST), (C) N1 duration, (D) N2 duration, (E) slow wave sleep (SWS) duration, (F) rapid eye movement (REM) sleep duration, (G) sleep efficiency, (H) wake after sleep onset (WASO), (I) N2 latency, and (J) mean slow wave activity (SWA) expressed as a percentage relative to baseline (B_2_). Shaded gray areas indicate the sleep manipulation periods. *** *p* < .001, ** *p* < .01, * *p* < .05 for group contrasts—red for stable short sleep group vs. control, blue for variable short sleep group vs. control, and purple for stable vs. variable short sleep groups.

Significant group × night interactions were found for all the sleep parameters—TIB, TST, N1, N2, SWS, REM, sleep efficiency, WASO, N2 latency, and mean SWA (*p* < .03, *ƒ*^2^ = 0.07–4666.89; Table 2). The negligible error bars for TIB (Figure 2A) indicated that there were minimal inter-individual differences within each group for all nights, and hence, our sleep opportunity manipulation was administered successfully. In this section, we will first describe the sleep physiological responses of each group across nights before reporting the group comparisons. While the results mainly present sleep measures in minutes, we have included the durations of each sleep stage as a percentage of TST in Supplementary Table S1 and reported the corresponding main and interaction effects in Supplementary Table S2.

**Table 2.** Main and interaction effects of group and night on sleep parameters.

	Main effect of group			Main effect of night			Interaction effect of group × night		
	*F*	*P*	*ƒ* ^2^	*F*	*P*	*ƒ* ^2^	*F*	*P*	*ƒ* ^2^
TIB	858 178.00	<.001	2799.93	192 036.00	<.001	4072.54	110 031.00	<.001	4666.89
TST	488.42	<.001	1.59	824.46	<.001	17.48	492.95	<.001	20.91
N1	2.60	.08	0.01	4.26	<.001	0.09	1.59	.03	0.07
N2	32.46	<.001	0.11	106.58	<.001	2.26	67.81	<.001	2.88
SWS	1.09	.34	0.004	3.35	<.001	0.07	2.13	<.01	0.09
REM	35.10	<.001	0.11	43.19	<.001	0.92	20.65	<.001	0.88
Sleep efficiency	10.78	<.001	0.04	4.60	<.001	0.10	1.70	.02	0.07
WASO	3.70	.03	0.01	5.00	<.001	0.11	1.71	.02	0.07
N2 latency	19.16	<.001	0.06	6.06	<.001	0.13	3.81	<.001	0.16
Mean SWA	10.14	<.001	0.03	16.28	<.001	0.35	8.20	<.001	0.35

REM, rapid eye movement; SWA, slow wave activity; SWS, slow wave sleep; TIB, time-in-bed; TST, total sleep time; WASO, wake after sleep onset.

### Control group

Provided with an 8-h TIB nightly, the control group’s TST remained relatively stable between 432.4 and 456.5 min throughout the protocol (Figure 2B). There were slight decreases in TST of about 10.2–19.8 min during the last three nights as compared to baseline (*p* < .03). These were accompanied by slight increases of 6.7–10.8 min in N2 latency (*p* < .05; Figure 2I) and decreases of 2.1%–4.1% in sleep efficiency (*p* < .05; Figure 2G) relative to baseline. Duration of N2, SWS, REM, and WASO, however, remained largely consistent throughout the 15 nights (*p* > .06; Figure 2, D–F and H), suggesting that the drops in sleep efficiency in the last three nights were mainly driven by the increased delays in sleep onset. Mean SWA remained mostly stable throughout the protocol (*p* > .06; Figure 2J). While there were a few isolated deviations from baseline levels in several of the sleep parameters (e.g. a small dip in mean SWA on R_1_2, *p* = .002, and fluctuations in N1 durations), these changes were non-systematic.

### Stable short sleep group

During the sleep manipulation nights, participants in the stable short sleep group received 6-h TIBs, which led to significant reductions in their TST to 345.3–351.2 min as compared to 454.8 min at baseline (*p* < .001; Figure 2B). N1, N2, and REM durations, as well as WASO, were also significantly shorter during the sleep-restricted nights as compared to baseline (*p* < .05; Figure 2, C, D, F, and H). Reductions in N2 and REM durations were largely proportional to reductions in TST (Supplementary Table S1). During the first sleep recovery period, TST, along with N2 and WASO, returned to baseline levels by the first recovery night R_1_1 (*p* > .14), while N1 and REM were at baseline levels by the second recovery night R_1_2 (*p* > .07). During the second sleep recovery period, all five sleep parameters were restored to baseline levels with just one night of recovery (R_2_1, *p* > .23). SWS duration was preserved throughout the protocol (*p* > .06), which can also be seen through the significant increase in the percentage of SWS on sleep-restricted nights as compared to baseline (*p* < .01; Supplementary Table S1). N2 latency showed steady decreases over the first few nights of sleep restriction in each cycle and was significantly shorter by M_1_2 (*p* = .003) and M_2_3 (*p* = .02) as compared to baseline (Figure 2I). However, no further drops in N2 latency were observed for the remaining sleep-restricted nights (M_1_2 vs. M_1_3–M_1_5: *p* > .28, M_2_3 vs. M_2_4–M_2_5: *p* > .63) and N2 latency returned to baseline levels by the first recovery night in both cycles (*p* > .11). Crucially, it should be noted that the changes in sleep macrostructure parameters observed during both sleep restriction periods were similar.

Mean SWA was significantly higher than baseline levels during the first sleep restriction period (*p* < .03), increasing significantly on M_1_1 because of the 2-h increase in the prior waking period and staying elevated for the remaining four nights (Figure 2J). It returned to baseline levels during the first recovery period (*p* > .06). However, while it showed some increase during the second sleep restriction period as well, mean SWA was still largely comparable to baseline levels for most nights (B_2_ vs. M_2_1–M_2_4: *p* > .20). The less prominent increase in SWA in the second week should not be taken as a sign that adults can adapt to recurrent periods of sleep restriction, because all the other sleep parameters (TST, N1, N2, SWS, REM, N2 latency, WASO, and sleep efficiency) did not show any significant differences between corresponding nights in the first and second sleep restriction periods (e.g. M_1_1 vs. M_2_1, M_1_2 vs. M_2_2, etc.) (*p* > .12), with the sole exception of REM duration between M_1_1 and M_2_1 (*p* = .03). Furthermore, mean SWA itself was only significantly different between M_1_1 and M_2_1 (*p* = .006) and not for the remaining manipulation nights (*p* > .05).

### Variable short sleep group

Participants in the variable short sleep group showed fluctuations in TST that were in line with the amount of TIB they received each night: TST was significantly reduced to 229.7–232.4 min during the 4-h TIB nights (*p* < .001) and 346.3–348.6 min during the 6-h TIB nights (*p* < .001), while it remained at around 452.8–462.0 min during the 8-h TIB nights (*p* > .18; Figure 2B). N1, N2, REM, and N2 latency also followed a similar pattern throughout the protocol. For example, N2 duration decreased significantly from baseline levels during the 4-h TIB on M_1_2 (*p* < .001, Figure 2D), returned to baseline during the 8-h TIB on M_1_3 (B_2_ vs. M_1_3: *p* = .51), before dropping again during the 4-h TIB on M_1_4 (*p* < .001). During the 6-h TIB on M_1_5, N2 duration increased to a level that was in between TIBs of 4 and 8 h. During the first recovery period, N2 returned to baseline levels (*p* > .22). The same pattern of fluctuation in N2 was repeated in the second cycle of sleep manipulation and recovery. WASO also showed a similar but muted fluctuation pattern throughout the protocol, such that the only significant deviations from baseline happened on M_1_2 and M_1_4 (*p* < .03; Figure 2H). Interestingly, there was a significant decrease in SWS from baseline levels on all 4-h TIB nights (*p* < .02; Figure 2E). While this finding could in part be due to the slightly longer SWS duration of this group at baseline relative to the control and stable short sleep groups, the prominent increase in the variable short sleep group’s SWS during the next sleep opportunity of 8 or 6 h (*p* < .01) points to the possibility that SWS was curtailed during the 4-h TIB nights; SWS durations on 8-h TIB and 6-h TIB nights were largely preserved (*p* > .09), except for M_2_1 (B_2_ vs. M_2_1: *p* = .01). Sleep efficiency remained relatively stable throughout the protocol, deviating from baseline only on M_1_5 (*p* = .047; Figure 2G).

Mean SWA also fluctuated according to TIB and hence, prior wake duration, showing the greatest increases from baseline on 4-h TIB nights (*p* < .001; Figure 2J), smaller increases on 6-h TIB nights (*p* < .02), and returning to baseline levels on all 8-h TIB nights (*p* > .12).

Similar to what was found in the stable short sleep group, the variable short sleep group showed comparable changes in the various sleep parameters during the two sleep manipulation periods. Significant differences between corresponding nights in the first and second sleep manipulation periods were found only for N2 and SWS, and only between M_1_1 and M_2_1 (N2: *p* = .02, SWS: *p* = .03), as well as M_1_3 and M_2_3 (N2: *p* = .01, SWS: *p* = .049).

### Group differences

As for between-group comparisons, there were no significant baseline differences in the sleep parameters (*p* > .09), apart from SWS duration. Despite the initial baseline difference in SWS duration between the variable short sleep group and the other two groups (vs. control: *p* = .004, vs. stable short sleep group: *p* = .04), by the following night M_1_1 when the variable short sleep group and the control group both had an 8-h TIB, the difference in SWS was no longer observed (*p* = .61; Figure 2E).

During the sleep-restricted nights, TST, N2, and REM in both short sleep groups showed dose-dependent reductions and were thus significantly shorter in duration as compared to the control group (*p* < .04; Figure 2, B, D, and F). N2 latencies in both short sleep groups were also significantly shorter than the control group on most sleep manipulation nights (*p* < .05; Figure 2I). N2 latencies between the two short sleep groups were also largely similar across all nights (*p* > .11), except for M_1_1 and M_2_1 (*p* < .05) which were nights when TIB was reduced for the stable but not variable short sleep group. Sleep efficiency was significantly higher in the two short sleep groups relative to the controls for most of the sleep manipulation nights (*p* < .05; Figure 2C). The only significant difference in sleep efficiency between the two short sleep groups occurred on M_1_2; however, the difference was small (1.7% ± 0.8%, *p* = .04). Mean SWA was significantly higher in both short sleep groups compared to the control group (*p* < .04, Figure 2J), excluding nights M_2_1 and M_2_4 (stable short sleep vs. control: *p* > .10). Group contrasts in the average values derived across each sleep restriction period showed that there were no significant differences between the two short sleep groups for any of the sleep parameters during both the first and second sleep restriction periods (*p* > .25).

During the first recovery period (R_1_1–R_1_2), there were no statistically significant group differences in the sleep parameters (*p* > .05), except on R_1_1 when the variable short sleep group had a significantly shorter REM duration than the control group (*p* = .04; Figure 2F), and the stable short sleep group had a significantly shorter N2 latency than the control group (*p* = .02; Figure 2I). Group contrasts in the average values derived across the first sleep recovery period showed that there were no significant differences between the two short sleep groups for any of the sleep parameters (*p* > .19). During the second recovery period, TST in both short sleep groups was significantly longer than in the control group (*p* < .001; Figure 2B). Correspondingly, sleep efficiency was also significantly higher (*p* < .002, Figure 2G). N2 latency was significantly shorter in the stable short sleep group than the control group (*p* = .006; Figure 2I), while WASO was significantly shorter in both short sleep groups compared to the control group (*p* < .03; Figure 2H). WASO was also significantly longer in the stable short sleep group than the variable short sleep group (*p* = .02).

## Discussion

The present study demonstrated that young adults subjected to successive cycles of moderate sleep restriction on weekdays and recovery sleep on weekends did not show significant differences in their sleep physiological responses between the first and second week. Moreover, differences in the sleep duration variability in the short sleep schedules did not appear to have any additional impact on the changes in sleep architecture.

### Changes to sleep architecture during a single cycle of sleep restriction and recovery

Focusing on just the first cycle of the study protocol, our findings showed that multiple nights of sleep restriction resulted in changes in sleep architecture that were largely consistent with previous research [6, 10, 11, 13–16]. In both short sleep groups, there were dose-dependent decreases in N1, N2, and REM sleep durations that matched the reductions in TIB. Increases in homeostatic sleep pressure from the longer prior wake durations were reflected by the shorter N2 latency and greater SWA on most of the sleep-restricted nights. Interestingly, while SWS was preserved during the 6-h TIB nights, it was significantly reduced on the 4-h TIB nights relative to baseline. This contrasts with previous studies [10, 14, 15, 20] that found that SWS durations remained relatively unchanged from baseline levels when TIB was restricted by a similar amount. In the study by Belenky et al., SWS was still preserved even during 3-h TIB nights [6]. The delayed bedtimes utilized in our study during the 4-h TIB nights may partially explain the SWS reductions: REM sleep undergoes stronger circadian promotion in the late biological night/early biological morning [37, 38] and therefore may have had disrupted SWS maintenance. However, it is unclear as to why we observed reductions in SWS on the 4-h TIB nights while other studies did not, particularly when comparing with studies that employed the same sleep restriction method of delaying bedtimes while keeping wake times constant [6, 10]. A possible explanation for the significant drop in SWS would be that baseline level of SWS for the variable short sleep group was somehow elevated; SWS duration at baseline was significantly longer in the variable short sleep group as compared to the other two groups. However, SWS in the variable short sleep group quickly rebounded back to baseline levels during the subsequent night when TIB was lengthened to either 6 or 8 h, instead of remaining at similar durations as the 4-h TIB nights, suggesting that 4-h TIB had indeed shortened SWS in our sample.

In line with prior sleep restriction studies in adults [6, 11, 13, 15], our findings also showed that the various sleep parameters affected by partial sleep loss (TST, N1, N2, REM, and N2 latency) all readily returned to baseline levels by the first or second night of sleep extension. For the stable short sleep group, this occurred during the first sleep recovery period (R_1_1–R_1_2), whereas for the variable short sleep group, this also occurred during the mid-week 8-h TIB night (M_1_3). There appears to be an age-related difference in the time needed for recovery of sleep physiology, with our previous adolescent studies showing that even up to three nights of sleep extension was insufficient for full recovery after multiple nights of sleep restriction [18, 19]. These contrasting findings highlight the need to investigate and directly compare the sleep recovery dynamics of various age groups (e.g. young children and elderly) following multi-night sleep curtailment.

### Repeated cycles of sleep restriction did not lead to cumulative effects on sleep architecture

In our previous study, the incomplete recovery for adolescents over the weekend recovery sleep period led to residual effects of sleep loss from the first week carrying over into the second week [19]. However, with full recovery achieved in young adults within just one or two 8-h TIB nights, it is perhaps unsurprising that in both short sleep groups, we generally did not observe significant differences between the first and second periods of sleep restriction for the various sleep parameters. This corresponds with Banks et al.’s study, which found that TST, N2, and REM sleep durations, as well as slow wave energy, remained stable across two recurrent cycles of 5 nights of 4-h TIB that was separated by an intervention night with TIB ranging from 0 to 12 h [20]. Our findings are also consistent with Van Dongen et al.’s study demonstrating that 14 days of sleep restriction to 4- or 6-h TIB did not result in significant cumulative changes in sleep architecture [10]. We found little evidence to suggest that homeostatic sleep drive accumulates across nights or across recurrent cycles of sleep restriction. That complete recovery was also achieved within the first night of recovery sleep after the second sleep restriction period provides further support to this claim.

Using the neurobehavioral data from the same study, we have previously reported that during recurrent cycles of sleep restriction, the stable short sleep group reported elevated but steady levels of subjective sleepiness across both sleep restriction periods [8], closely matching the changes observed in their SWA levels in the preceding nights. However, we also found that vigilance deficits accumulated with more nights of sleep restriction and even compounded during the second sleep restriction period. This cumulative deterioration in vigilance cannot be accounted for by the changes in sleep architecture. The disparity between sleep physiological responses and waking neurobehavioral responses to repeated sleep restriction has also been reported by Van Dongen et al. [10] and Banks et al. [20] who restricted their adult participants to a greater extent (i.e. 4 h per night). These consistent findings across studies suggest that sleep and neurobehavioral responses during multi-night partial sleep deprivation may be regulated by distinct physiological mechanisms.

### Sleep duration variability had minimal effect on sleep architecture response

In this study, we extended previous findings by demonstrating that sleep duration variability showed minimal to no effect on sleep architecture in short sleep schedules, largely similar to what was found in prior studies investigating schedules with average TIBs within the recommended 7–9 h [23, 24]. Specifically, no significant differences were found between the two short sleep groups for any of the sleep parameters when averaging across each period of sleep restriction or recovery. One noticeable difference was that Taub [22] and Sun et al. [23] found greater sleep duration variability to be associated with more average N2 sleep. This difference could be dependent on whether participants were, overall, sleep-restricted (current study) or had TIBs within the age-appropriate range (previous studies). Our findings that increasing night-to-night variability in sleep duration would not minimize, or aggravate, changes in sleep architecture resulting from sleep restriction indicate that neither short sleep schedules would be a better option for sleep-restricted individuals in terms of sleep physiology, highlighting the importance of getting adequate sleep on a consistent basis.

### Limitations and future studies

There were a few limitations to our study. Our sleep manipulation was achieved by delaying bedtimes while keeping wake times constant throughout the protocol. This may have resulted in circadian phase delays [39], which could lead to different results when TIB was shortened by advancing wake times or altering both bedtimes and wake times to align the mid-points of the sleep episodes. Our study also only included one example of a variable sleep schedule (i.e. 8-h, 4-h, 8-h, 4-h, and 6-h TIBs across five nights). Future studies should investigate whether sleep schedules with greater or smaller sleep duration variability, or even whether reordering the TIBs across nights, would lead to similar findings, e.g. similar sleep responses in the first and second weeks of sleep curtailment, and between individuals having the same sleep–wake timings across nights and those adopting a more variable sleep schedule. It would also be interesting to examine whether our findings would be replicated in other age groups. For example, would adolescents following a more variable short sleep schedule still show compounding effects in the second sleep restriction period, similar to those following a stable short sleep schedule in our previous study [19]? Furthermore, while our study intentionally implemented strict inclusion criteria to ensure that any effects observed could be reliably attributed to our experimental manipulation, it also resulted in a sample of healthy young adults who may be more resilient to repeated sleep restriction than the general population and thus may limit the generalizability of our findings. Future studies should include a more representative sample of the general population to determine if our findings can be replicated. Lastly, we mainly used C3, F3, and O1 in the PSG montage for this study, and thus it may be possible that if there were regional differences in brain activities caused by our experimental manipulation, we would not have been able to detect them.

## Conclusion

The present study has shown that in young adults, changes in sleep architecture induced by multiple nights of moderate partial sleep loss can recover quickly and that re-exposure to another cycle of sleep restriction results in similar alterations in sleep architecture. Also, a more variable short sleep schedule showed little additional impact on changes in sleep architecture when compared to a stable short sleep schedule.

## Supplementary Material

zpaf016_suppl_Supplementary_Material

## Data Availability

The data underlying this article will be shared on reasonable request to the corresponding author.
