# Supplementary material for: Changes in sleep architecture during recurrent cycles of sleep restriction: a comparison between stable and variable short sleep schedules
Source: Sleep Adv. 2025 Mar 15;6(2):zpaf016. doi: 10.1093/sleepadvances/zpaf016 (PMC12084804; doi:10.1093/sleepadvances/zpaf016)
Supplement: zpaf016_suppl_Supplementary_Material [file zpaf016_suppl_supplementary_material.docx]

**Supplementary Material**

**Changes in sleep architecture during recurrent cycles of sleep restriction: a comparison between stable and variable short sleep schedules**

Tiffany B. Koa^1^; Ju Lynn Ong^1^; June C. Lo^1^

^1^Centre for Sleep and Cognition and Human Potential Translational Research Programme, Yong Loo Lin School of Medicine, National University of Singapore, Singapore

Corresponding author:

Dr. June Chi-Yan Lo

Centre for Sleep and Cognition

Yong Loo Lin School of Medicine,

National University of Singapore,

MD1 Tahir Foundation Building, 12 Science Drive 2,

Singapore 117549

Phone: (+65) 66016146

E-mail: june.lo@nus.edu.sg

**Supplemental Material**

Table S1. Durations of each sleep stage presented as percentages of total sleep time for each group.

|  | **Protocol night** | | | | | | | | | | | | | |
| --- | --- | --- | --- | --- | --- | --- | --- | --- | --- | --- | --- | --- | --- | --- |
|  | **B_2_** | **M_1_1** | **M_1_2** | **M_1_3** | **M_1_4** | **M_1_5** | **R_1_1** | **R_1_2** | **M_2_1** | **M_2_2** | **M_2_3** | **M_2_4** | **M_2_5** | **R_2_1** |
| **Control** | |  |  |  |  |  |  |  |  |  |  |  |  |  |
| N1 (% TST) | 1.25  (0.19) | 0.67  (0.20) | 1.01  (0.20) | 1.27  (0.21) | 0.60  (0.21) | 0.66  (0.22) | 1.02  (0.23) | 0.89  (0.23) | 0.66  (0.24) | 0.84  (0.24) | 1.07  (0.25) | 1.16  (0.26) | 0.91  (0.28) | 0.93  (0.28) |
| N2 (% TST) | 50.60  (1.26) | 47.81  (1.34) | 50.70  (1.35) | 50.86  (1.41) | 49.71  (1.47) | 49.65  (1.58) | 48.47  (1.66) | 49.29  (1.74) | 49.52  (1.83) | 52.24  (1.88) | 51.28  (1.98) | 48.93  (2.08) | 50.13  (2.24) | 48.72  (2.30) |
| SWS (% TST) | 17.51  (0.99) | 19.63  (1.07) | 17.50  (1.10) | 17.33  (1.17) | 18.08  (1.25) | 17.60  (1.36) | 17.93  (1.44) | 17.15  (1.54) | 16.84  (1.63) | 17.09  (1.71) | 17.13  (1.81) | 16.98  (1.92) | 16.70  (2.06) | 16.62  (2.15) |
| REM (% TST) | 24.70  (0.97) | 26.47  (1.02) | 25.10  (1.01) | 24.73  (1.04) | 25.33  (1.07) | 25.46  (1.13) | 27.54  (1.17) | 26.93  (1.21) | 24.71  (1.25) | 24.56  (1.27) | 23.88  (1.32) | 24.72  (1.37) | 24.22  (1.48) | 23.70  (1.50) |
| **Stable Short Sleep** | |  |  |  |  |  |  |  |  |  |  |  |  |  |
| N1 (% TST) | 1.35  (0.17) | 1.04  (0.18) | 0.66  (0.18) | 0.93  (0.18) | 0.61  (0.19) | 0.600  (0.21) | 0.82  (0.20) | 0.97  (0.20) | 0.62  (0.21) | 0.63  (0.22) | 0.42  (0.22) | 0.59  (0.23) | 0.62  (0.24) | 1.18  (0.25) |
| N2 (% TST) | 51.56  (1.12) | 48.28  (1.16) | 48.67  (1.23) | 47.50  (1.25) | 49.22  (1.31) | 48.02  (1.45) | 50.81  (1.44) | 52.48  (1.52) | 47.27  (1.59) | 48.67  (1.72) | 49.10  (1.76) | 48.10  (1.86) | 47.73  (1.95) | 51.73  (2.04) |
| SWS (% TST) | 18.66  (0.88) | 24.27  (0.92) | 23.78  (1.00) | 25.18  (1.04) | 23.68  (1.11) | 24.63  (1.24) | 18.56  (1.26) | 17.00  (1.35) | 22.74  (1.43) | 24.07  (1.55) | 22.80  (1.61) | 23.72  (1.72) | 25.15  (1.81) | 17.42  (1.91) |
| REM (% TST) | 23.03  (0.86) | 22.09  (0.88) | 24.03  (0.92) | 22.39  (0.92) | 22.56  (0.95) | 23.34  (1.05) | 25.33  (1.01) | 25.01  (1.05) | 25.20  (1.09) | 22.62  (1.17) | 24.24  (1.17) | 24.20  (1.24) | 22.66  (1.28) | 23.59  (1.33) |
| **Variable Short Sleep** | |  |  |  |  |  |  |  |  |  |  |  |  |  |
| N1 (% TST) | 0.95  (0.18) | 0.94  (0.18) | 0.47  (0.18) | 0.73  (0.19) | 0.39  (0.19) | 0.52  (0.20) | 0.55  (0.22) | 0.67  (0.21) | 0.91  (0.22) | 0.34  (0.23) | 0.70  (0.23) | 0.58  (0.24) | 0.43  (0.25) | 0.69  (0.25) |
| N2 (% TST) | 49.81  (1.18) | 48.30  (1.19) | 41.19  (1.23) | 48.90  (1.29) | 40.92  (1.35) | 46.55  (1.41) | 48.92  (1.60) | 50.85  (1.58) | 51.74  (1.64) | 41.40  (1.74) | 53.06  (1.81) | 38.99  (1.90) | 47.73  (1.99) | 50.68  (2.08) |
| SWS (% TST) | 20.87  (0.93) | 20.24  (0.95) | 36.29  (1.00) | 22.53  (1.07) | 32.86  (1.14) | 27.85  (1.22) | 21.22  (1.38) | 18.77  (1.40) | 17.65  (1.47) | 33.85  (1.58) | 20.16  (1.66) | 33.81  (1.75) | 27.63  (1.85) | 19.45  (1.95) |
| REM (% TST) | 23.34  (0.91) | 24.73  (0.90) | 17.50  (0.92) | 23.05  (0.95) | 21.91  (0.98) | 21.73  (1.01) | 24.72  (1.14) | 24.53  (1.10) | 24.35  (1.12) | 20.77  (1.18) | 22.19  (1.21) | 22.54  (1.25) | 20.16  (1.30) | 23.52  (1.35) |

REM, rapid eye movement; SWS, slow wave sleep; TST, total sleep time. Estimates of means and standard errors were derived from general linear mixed models.

Table S2. Main and interaction effects of group and night on sleep stages expressed as percentages of total sleep time.

|  | **Main effect of group** | | | **Main effect of night** | | | **Interaction effect of group x night** | | |
| --- | --- | --- | --- | --- | --- | --- | --- | --- | --- |
|  | **F** | ***p*** | ***ƒ*^2^** | **F** | ***p*** | ***ƒ*^2^** | **F** | ***p*** | ***ƒ*^2^** |
| N1 (% TST) | 1.37 | .26 | 0.004 | 2.62 | < .01 | 0.06 | 1.01 | .45 | 0.04 |
| N2 (% TST) | 1.70 | .18 | 0.01 | 7.06 | < .001 | 0.15 | 6.19 | < .001 | 0.26 |
| SWS (% TST) | 12.19 | < .001 | 0.04 | 31.98 | < .001 | 0.68 | 18.63 | < .001 | 0.79 |
| REM (% TST) | 3.66 | .026 | 0.01 | 4.14 | < .001 | 0.09 | 1.91 | < .001 | 0.08 |

REM, rapid eye movement; SWS, slow wave sleep; TST, total sleep time.
